# Supplementary material for: Psychiatric inpatient care for persons with dissociative identity disorder: a scoping review protocol
Source: BMJ Open. 2024 Feb 15;14(2):e079207. doi: 10.1136/bmjopen-2023-079207 (PMC10973692; doi:10.1136/bmjopen-2023-079207)
Supplement: Supplementary data [file bmjopen-2023-079207supp001.pdf]

Supplemental table 1. Data charting table

Authors	Year of publication	Country	Population	Setting	Aim	Design	Discipline	Quality	RQ1	RQ2	RQ3	RQ4	RQ5
