# Supplementary material for: Psychiatric inpatient care for persons with dissociative identity disorder: a scoping review protocol
Source: BMJ Open. 2024 Feb 15;14(2):e079207. doi: 10.1136/bmjopen-2023-079207 (PMC10973692; doi:10.1136/bmjopen-2023-079207)
Supplement: Author's manuscript [file bmjopen-2023-079207.draft_revisions.pdf]

BMJ Open is committed to open peer review. As part of this commitment we make the peer review history of every article we publish publicly available.

When an article is published we post the peer reviewers' comments and the authors' responses online. We also post the versions of the paper that were used during peer review. These are the versions that the peer review comments apply to.

The versions of the paper that follow are the versions that were submitted during the peer review process. They are not the versions of record or the final published versions. They should not be cited or distributed as the published version of this manuscript.

BMJ Open is an open access journal and the full, final, typeset and author-corrected version of record of the manuscript is available on our site with no access controls, subscription charges or pay-per-view fees (http://bmjopen.bmj.com).

If you have any questions on BMJ Open's open peer review process please email info.bmjopen@bmj.com

BMJ Open

Psychiatric inpatient care for persons with dissociative identity disorder: A scoping review protocol

Journal:	*BMJ Open*
Manuscript ID	bmjopen-2023-079207
Article Type:	Protocol
Date Submitted by the Author:	25-Aug-2023

Complete List of Authors:	Söderberg, Anja; Lulea University of Technology Department of Health Education and Technology,<br>Lindgren, Britt-Marie; Umea Universitet, Nursing<br>Ejneborn-Looi, Git-Marie; Luleå University of Technology, Department of Health Sciences<br>Bäckström, Josefin; Lulea University of Technology Department of Health Education and Technology<br>Gabrielsson, Sebastian; Luleå University of Technology, Department of Health Sciences

Keywords:	MENTAL HEALTH, PSYCHIATRY, Hospitals, Hospitalization

SCHOLARONE™ Manuscripts

Psychiatric inpatient care for persons with dissociative identity disorder: A scoping review protocol

Anja Söderberg[1] MSc, PhD Student, RN anja.soderberg@ltu.se

Britt-Marie Lindgren[2] PhD, Associate Professor, RN britt-marie.lindgren@umu.se

Git-Marie Ejneborn Looi[1] PhD, Senior Lecturer, RN git-marie.ejneborn-looi@ltu.se

Josefin Bäckström[1,3] PhD, Senior Lecturer, RN josefin.backstrom@ltu.se

Sebastian Gabrielsson1 PhD, Associate Professor, RN sebastian.gabrielsson@ltu.se

[1]Department of Health, Education and Technology, Luleå university of technology

[2]Department of Nursing, Umeå university

[3] Department of Medical Sciences, Psychiatry, Uppsala University

Corresponding author:

Anja Söderberg

Department of Health, Education and Technology

Luleå University of Technology

971 87 Luleå

Sweden

anja.soderberg@ltu.se

For peer review only

For peer review only - http://bmjopen.bmj.com/site/about/guidelines.xhtml

Abstract

Introduction

Psychiatric inpatient care is often characterized by high pressure and thresholds for admission, brief periods of care and limited time for caring activities. Dissociative identity disorder is a contested diagnosis and persons with dissociative identity disorder are at risk of not receiving adequate support when cared for in psychiatric inpatient care. Because the limited literature addressing the topic includes no overview on how persons with dissociative identity disorder are cared for in psychiatric inpatient settings, the aim of this proposed scoping review is to map the area of knowledge on psychiatric inpatient care for persons experiencing dissociative identity disorder. This proposed scoping review will provide an overview with the possibility to elucidate gaps in the evidence base and needs for future research on psychiatric inpatient care for persons experiencing dissociative identity disorder.

Methods and analysis

This proposed scoping review will follow Preferred Reporting Items for Systematic Review and Meta-Analysis for Scoping Reviews and steps 1-5 described in established method for scoping reviews: identifying research question, identifying relevant studies, study selection, charting the data and collating, summarizing and reporting results.

Ethics approval

Not applicable

Dissemination

This proposed scoping review will be submitted for publication in an international, peer-reviewed journal.

For peer review only - http://bmjopen.bmj.com/site/about/guidelines.xhtml

Keywords

Psychiatric inpatient care, dissociative identity disorder, DID, scoping review

Strengths and limitations

The strength with this proposed scoping review is that it will map the area of knowledge in a broad sense, including different types of publications. It will not, however, synthesize the findings which is a limitation. The lack of quality assessment is a known limitation for scoping reviews but in this proposed scoping review the quality assessment will be a part of the data charting which is considered to be a strength.

Introduction

Persons with experience of DID and other severe dissociative states following trauma experience represent a vulnerable group in PIC. This calls for contemporary, personcentered approaches to care that prevent retraumatization and further harm [1]. In those efforts, awareness of the importance of traumainformed care (TIC) in psychiatric inpatient settings has increasingly been raised [2, 3, 4, 5, 6]. The focus for this proposed scoping review is to provide an overview of what is known about PIC for persons experiencing DID.

Background

PIC is a form of care in which the person is cared for 24 hours a day and the daily activities are regulated by staff [7]. Following the widespread deinstitutionalization in the second half of the 20th century, major changes have been made in the organization of psychiatric care around the world [8], such that PIC is no longer the dominant form of care [7]. Access to PIC

For peer review only - http://bmjopen.bmj.com/site/about/guidelines.xhtml

For peer review only

varies worldwide, from less than two to over 25 hospital beds per 100 000 population [9]. McCrae [10] argues that along with the organizational changes, the view on PIC has also shifted to the idea that PIC in itself is something bad that should be avoided at all costs. Beyond that, PIC settings are often characterized by high pressure and thresholds for admission, brief periods of care and limited time for caring activities [10]. Even so, PIC is considered to be necessary for persons with acute psychiatric problems [11] and PIC of good quality is described to be based on relationships and moreover to be caring, person centered and recovery oriented [12]. Despite recommended models, including TIC, developed to improve the quality of care in PIC [2, 3, 4, 5, 6], PIC facilities struggle to embrace a coherent vision and goal for the delivery of care [13].

Dissociative states, among which DID is considered to be the most severe type [14], include symptoms of depersonalization, derealization [15], flashbacks, nightmares and switching between separate parts of the identity through compartmentalization [16]. Stigma is recognized to affect persons with dissociative states, who may be undiagnosed for several years and feel shame about their experiences of dissociation [14]. DID is also known to be a controversial and questioned diagnosis [14, 16, 17, 18] with symptoms that can be misinterpreted as better known diagnosis such as schizophrenia or personality syndrome [14, 16, 18] and the person with DID is at risk of not getting adequate treatment and being met with disbelief [1, 18].

Persons experiencing DID are at great risk of self injury and suicide attempts [1] and may require PIC in order to ensure their personal safety and a stabilized wellbeing [14]. There is reason to believe that people with DID would benefit from contemporary developments of PIC, including personcentered, recovery-oriented and traumainformed approaches to care [cf. 1]. To date, research on PIC for persons with DID is limited and without any coherent description on the experiences and impact of PIC for persons with DID cared for in PIC. In

For peer review only - http://bmjopen.bmj.com/site/about/guidelines.xhtml

response, this proposed scoping review will provide an overview with the possibility to elucidate gaps in the evidence base and needs for future research. Due to the relatively unexplored area, a descriptive design is motivated and this proposed scoping review is aiming to map the area of knowledge on psychiatric inpatient care for persons with DID, striving to be as comprehensive as possible in order to identify all relevant literature regardless of study design. The method of scoping review is suitable due to its ability to address broad research questions, map relevant literature and to elucidate the field of interest [cf 21].

Methods and analysis

This scoping review protocol follows Preferred Reporting Items for Systematic Review and Meta-Analysis Protocols checklist (PRISMA-P) [cf 19] and the proposed scoping review will follow Preferred Reporting Items for Systematic Review and Meta-Analysis for Scoping Reviews (PRISMA-ScR) [cf 20].

Step 1-5 in the established method for scoping reviews described by Arksey and O'Malley [21] will be performed as described in what follows:

Identifying research questions

This proposed scoping review aims to map the area of knowledge on PIC for persons with DID. In specific, the review seeks to explore the following research questions (RQ):

- RQ1: How is DID conceptualized in the context of PIC?
- RQ2: Why are persons with DID subject to PIC?
- RQ3: What mechanisms of PIC for persons with DID are described?
- RQ4: What experiences of PIC for persons with DID are described?
- RQ5: What outcomes of PIC for persons with DID are described?

For peer review only - http://bmjopen.bmj.com/site/about/guidelines.xhtml

Identifying relevant studies

To be as comprehensive as possible, the search strategy will include both peer reviewed papers published in international scholarly journals indexed in curated databases and grey literature [cf 21]. For the purpose of this proposed review, grey literature refers to research not intended for publication, or not yet published, in international scholarly journals, i.e. dissertations, thesis, conference publications, unpublished manuscripts, clinical guidelines and reports from governmental or non-governmental organizations.

<u>Search strategy</u>

The first author will perform all of the described steps of the search strategy.

Curated databases

Consultations with a librarian have been undertaken and pilot studies have been performed in order to refine the search strategy. The databases PubMed, Cinahl and PsycINFO have been identified as being suitable for this proposed scoping review, related to the research area. Key concepts related to the aim and the research questions have also been identified and will form the search blocks. In the search blocks, subject headings will be used together with related terms in free text, and the Boolean operators ("AND" and "OR") will be used to narrow and expand the search. Each search in the databases will be documented with date, search terms, number of search matches, selected studies and limitations. The search strategy for each chosen database is described in Table 1, followed by an example of a pilot search (Table 2):

For peer review only - http://bmjopen.bmj.com/site/about/guidelines.xhtml

Table 1: Search strategy			
**Key concept**	**PubMed**	**Cinahl**	**PsycINFO**
DID	(dissociative identity disorder [MeSH] OR "multiple personality disorder")	("dissociative identity disorder" OR multiple personality disorder [MH] OR did OR mpd)	(dissociative identity disorder [DE] OR "multiple personality disorder")
Psychiatry	(psychiatry [MeSH] OR psychiatric OR mental health [MeSH] OR "mental illness" OR mental disorders [MeSH])	(psychiatry [MH] OR psychiatric OR mental health OR "mental illness" OR mental disorders [MH])	(psychiatry [DE] OR psychiatric OR mental health [DE] or "mental illness" [DE] OR mental disorders [DE])
Inpatient care	(inpatients [MeSH] OR ward OR hospital* OR "acute setting")	(inpatients [MH] OR ward OR hospital* OR "acute setting")	(inpatients OR ward OR hospital* OR "acute setting")

Table 2: Pilot search		
PubMed 230104		
*Limitations: Published between 2000-2022		
Search	*Search terms*	*Matches*
S1	exp dissociative identity disorder/	1 186
S2	"multiple personality disorder".mp	356
S3	S1 or S2	1 218
S4	exp psychiatry/	760 502
S5	psychiatric.mp	937 395
S6	exp mental health/	487 022
S7	"mental illness".mp	40 185
S8	exp mental disorders/	1 459 534
S9	S4 or S5 or S6 or S7 or S8	2 079 336
S10	exp inpatients/	158 439
S11	ward.mp	101 353
S12	hospital*.mp	6 577 438
S13	"acute setting".mp	2 637
S14	S10 or S11 or S12 or S13	6 669 493
S15	S3 and S9 and S14	260
S16	S3 and S9 and S14*	120

Additional sources

Additional searches will be performed manually in Google Search and Google Scholar and possibly other databases as well. To further identify relevant studies that may not have been found by performing the search strategy in the electronic databases and the additional searches, the reference lists of selected studies will also be screened. Searches

For peer review only - http://bmjopen.bmj.com/site/about/guidelines.xhtml

for studies that have cited the selected studies will additionally be performed. Key journals, identified through the selected studies, will be searched manually to identify studies that may have been missed in the searches in the electronic databases, in the screening of reference lists and in the citation searches. Last, searches through existing knowledge and networks related to the research area will be performed to find information about, for example, studies that have not yet been published [cf 21].

Study selection

To determine the relevance of the studies found by performing the search strategy the inclusion and exclusion criteria will be applied to all the studies found by performing the search strategy. The aim of the inclusion and exclusion criteria is to keep eligible studies and exclude irrelevant studies [cf 21]. In this proposed scoping review the general inclusion and exclusion criteria will consist of year of publication, language and type of study, whereas other inclusion and exclusion criteria are formulated in accordance with the research questions (Table 3):

For peer review only

Table 3: Inclusion and exclusion criteria		
	**Inclusion**	**Exclusion**
Persons with DID	• Studies focused on persons with DID<br>• Studies with small samples of persons with DID but allow distinguishing persons with DID from the other population studied	• Studies focused on persons with DID and a comorbidity
Psychiatry	• Studies focused on psychiatric care	
Inpatient care	• Studies focused on any context in which the person is cared for 24 hours a day<br>• Studies with small samples of inpatient care setting but allow distinguishing the inpatient care setting from other settings studied	
Year of publication	• 2000 - 2023	
Language	• English<br>• Swedish<br>• Norwegian<br>• Danish	
Type of study	• Any paper reporting an original study<br>• Internationally published studies<br>• Grey literature formed as dissertations, thesis, conference publications, unpublished manuscripts, clinical guidelines and reports from governmental and non governmental organizations	• Editorials<br>• Discursive papers<br>• Literature reviews

For peer review only - http://bmjopen.bmj.com/site/about/guidelines.xhtml

Regarding year of publication, a longer period of time is chosen in order to gather enough data and has been considered in relation to the relatively unexplored area. The limit of the year 2000 has been set due to the organization of PIC after the deinstitutionalization.

To apply the inclusion and exclusion criteria to all of the identified studies, a systematic approach will be undertaken. The screening of all studies will follow a combined template for screening and charting (Table 4).

For peer review only

For peer review only - http://bmjopen.bmj.com/site/about/guidelines.xhtml

Table 4: Screening and charting template	
**Step 1: Title and abstract screening**	
1. Is this title and abstract written in English/Swedish/Norwegian/Danish?	Yes/No
2. Does it seemingly address DID in PIC?	Yes/No
**Step 2: Full-text screening**	
1. Is there any reason this article should be excluded?	Yes/No
If yes:<br>What is the reason for excluding?	1. Not in English/Swedish/Norwegian/Danish<br>2. Not published between 2000 – 2023<br>3. Not focus on DID<br>4. Not focus on PIC<br>5. No full text available despite efforts to retrieve
If no:<br>What is the data charting information?	Use the data charting table
2. Are there descriptions on how DID is conceptualized in the context of PIC (RQ1)?	Yes/No
If yes, describe how:	
3. Are there descriptions of why persons with DID are subject to PIC (RQ2)?	Yes/No
If yes, describe how:	
4. Are there descriptions of mechanisms of PIC for persons with DID (RQ3)?	Yes/No
If yes, describe how:	
5. Are there descriptions of experiences of PIC for persons with DID (RQ4)?	Yes/No
If yes, describe how:	
6. Are there descriptions of outcomes of PIC for persons with DID (RQ5)?	Yes/No
If yes, describe how:	

The results of the searches from each database will be downloaded in the screening tool Rayyan to support a systematic screening. A first selection of studies will be made through applying the inclusion criteria on the title and abstract of all the studies. The next step will include reading the studies in full in order to make a final decision whether to include or

For peer review only - http://bmjopen.bmj.com/site/about/guidelines.xhtml

exclude, which is required since abstracts can't be expected to represent the full study or to grasp the full scope of the study [cf 21]. Both steps of the study selection will include two authors independently screening the studies. If disagreements whether to include or exclude the two authors will discuss and try to reach consensus, and if needed a third author will be involved to resolve discrepancies. The process of selecting eligible studies will follow the Preferred Reporting Items for Systematic Review and Meta-Analysis (PRISMA) flow diagram (Figure 1) [22]:

Figure 1. PRISMA flow diagram

Charting the data

The combined template for screening and charting (Table 4) will also include steps for charting the data and will be used to support a systematic approach. Data from the selected studies will then be charted according to the research questions. The charted data will be transferred to a data charting table (Supplemental table 1) that includes the following information:

- Authors
- Year of publication
- Country
- Population
- Setting
- Aim
- Design
- Discipline
- Quality
- RQ1

For peer review only - http://bmjopen.bmj.com/site/about/guidelines.xhtml

- RQ2
- RQ3
- RQ4
- RQ5

The charted data will then be used as the foundation for analysis [cf 21]. Two authors will chart the data independently and disagreements will be resolved through discussion until consensus is reached.

<u>Quality assessment</u>

Because scoping reviews generally seek comprehensiveness, breadth and inclusion, a quality assessment is not included in the description of a scoping review which is acknowledged as a limitation of the method [21]. However, in this proposed scoping review a quality assessment will be performed as a part of the data charting in order to also provide an overview on the quality of the existing research. The Mixed Method Appraisal Tool [23] will be used for the quality appraisal of the found studies. This tool is developed to be used in reviews containing qualitative, quantitative and mixed method studies [23], why it's chosen for the quality appraisal of this review. Two authors will independently perform the quality appraisal and disagreements will be discussed until consensus is reached. A third author will be involved in the process to resolve discrepancies if needed.

Collating, summarizing and reporting the results

The final part of this proposed scoping review will provide an overview of the research by collating, summarizing and reporting results. One part of this analysis will be a basic numerical analysis of the included studies, meaning the data presented in the data charting table (Supplemental table 1), in order to bring light to dominant areas of research by to country, study population, setting, aims, designs and disciplines. The second part of the

For peer review only - http://bmjopen.bmj.com/site/about/guidelines.xhtml

analysis will be to organize the data thematically according to the research questions. A clear reporting strategy will be undertaken in order to determine potential bias, which is why a consistent approach based on the combined template for screening and charting (Table 4), also covering the research questions, will be used. The next step will be to make comparisons, identify contradictions, identify gaps in the evidence base and suggest topics for future research [cf 21]. All authors will be involved in the final discussion about the collated, summarized and reported results.

Patient and public involvement

A non-governmental organization advocating the interests of persons experiencing DID have been engaged in the development of the research questions and the design of the review. The findings from the review will be communicated with representatives of the organization.

List of abbreviations

**DID**	Dissociative identity disorder
**PIC**	Psychiatric inpatient care
**PRISMA**	Preferred reporting items for systematic review and meta-analysis
**PRISMA-P**	Preferred reporting items for systematic review and meta-analysis protocols
**PRISMA-ScR**	Preferred reporting items for systematic review and meta-analysis for scoping reviews
**TIC**	Traumainformed care

For peer review only - http://bmjopen.bmj.com/site/about/guidelines.xhtml

For peer review only

Ethics approval

Not applicable

Dissemination

This proposed scoping review will be submitted for publication in an international, peer-reviewed journal

Authors' contributions

AS, SG, BL and GEL have made substantial contributions to the conception and the design. AS and SG have drafted the work and AS, SG, BL, GEL and JB have substantively revised it. Each author has approved to the submitted version and have agreed to be personally accountable and to ensure that questions related to the accuracy or integrity of any part of the work, even ones in which the author was not personally involved, are appropriately investigated, resolved, and the resolution documented in the literature.

Funding

This research is funded by Luleå university of technology

Competing interests statement

The authors declare no conflict of interest

For peer review only - http://bmjopen.bmj.com/site/about/guidelines.xhtml

References

1. Parry, S., Lloyd, M. & Simpson, J. Experiences of therapeutic relationships on hospital wards, dissociation, and making connections. J Trauma Dissociatio. 2017;18(4): 544-558. https://doi.org/10.1080/15299732. 2016.1241852

2. Isobel, S. 'Because that's the way it's always been done': Reviewing the nurse-initiated rules in a mental health unit as a step toward trauma-informed care. Issues in Ment Health N. 2015;36: 272-278. https://doi.org/10.3109/01612840.2014.982842

3. Isobel, S. & Edwards, C. Using trauma informed care as a nursing model of care in an acute inpatient mental health unit: A practice development process. Int J Ment Health Nu. 2016. https://doi.org/10.1111/inm.12236

4. O'Dwyer, C., Tarzia, L., Fernbacher, S. & Hegarty, K. Health professionals' experiences of providing trauma-informed care in acute psychiatric inpatient settings: A scoping review. Trauma Violence Abus. 2021;22(5): 1057-1067. https://doi.org/10.1177/1524838020903064

5. Muskett, C. Trauma-informed care in inpatient mental health settings: A review of the literature. Int J Ment Hlt Nu. 2014;23: 51-59. https://doi.org/10.1111/inm.12012

For peer review only - http://bmjopen.bmj.com/site/about/guidelines.xhtml

6. Wilson, A., Hutchinson, M. & Hurley, J. Literature review of trauma-informed care: Implications for mental health nurses working in acute inpatient settings in Australia. Int J Ment Hlt Nu. 2017;26: 326-343. https://doi.org/10.1111/inm.12344

7. Topor, A., Andersson, G., Bülow, P., Stefansson, C. G., & Denhov, A. After the asylum? The new institutional landscape. Community Ment Hlt J. 2016;52(6): 731-737

8. Bergmark, M., Bejerholm, U. & Markström, U. (2017). Policy changes in community mental health: Interventions and strategies used in Sweden over 20 years. Soc Policy Admin. 2017;51: 95–113. https://doi.org/10.1111/spol.1275

9. World Health Organization. Mental Health Atlas 2020. World Health Organization; 2021.

10. McCrae, N. Lessons from a lost world: Messages from the mental hospital era. Editorial. J Adv Nurs. 2017;74(6): 1226-1228. https://doi.org/10.1111/jan.13467

11. Jacob, K. S., Sharan, P., Mirza, I., Garrido-Cumbrera, M., Seedat, S., Mari, J. J., Sreenivas, V. & Saxena, S. (2007). Mental health systems in countries: where are we now? Lancet. 2007; 370(9592), 1061-1077

For peer review only

For peer review only - http://bmjopen.bmj.com/site/about/guidelines.xhtml

12. Gabrielsson, S., Tuvesson, H., Wiklund Gustin, L. & Jormfeldt, J. Positioning psychiatric and mental health nursing as a transformative force in health care. Issues Ment Health N. 2020;41(11): 976-984. https://doi.org/10.1080/01612840.2020.1756009

13. Salberg, J., Bäckström, J., Röing, M., & Öster, C. Ways of understanding nursing in psychiatric inpatient care–A phenomenographic study. J Nurs Manage. 2019; 27(8): 1826-1834.

14. Snyder, B.L. Recognizing & treating dissociative disorders: the nurse's role in supporting patients and their families. American Nurse Journal. 2021;16(9): 2-5.

15. Hill, S.B., Wolff, J.D., Bigony, C.E., Winternitz, S.R., Ressler, K.J., Kaufman, M.L. & Lebois, L.A.M. Dissociative subtype of posttraumatic stress disorder in women in partial and residential levels of psychiatric care. J Trauma Dissociation. 2020; 21(3): 305-318. https://doi.org/10.1080/15299732.2019.1678214

16. McAllister, M. M. Dissociative identity disorder: A literature review. J Psychiatr Ment Hlt. 2000;7(1): 25-33. https://doi.org/10.1046/j.1365-2850.2000.00259

17. Stickley, T., & Nickeas, R. Becoming one person: Living with dissociative identity disorder. J Psychiatr Ment Hlt. 2006;13(2): 180-187.

For peer review only

18. Tomlinson, K. & Baker, C. Women's auto/biography and dissociative identity disorder: implications for mental health practice. Journal of Medical Humanit. 2019;40: 365-387. https://doi.org/10.1007/s10912-017-9471-3

19. Moher, D., Stewart, L. & Shekelle, P. Implementing PRISMA-P: recommendations for prospective authors. Syst Rev. 2016;5(15). https://doi.org/10.1186/s13643-016-0191-y

20. Tricco, A.C., Lillie, E., Zarin, W. et al. PRISMA extension for scoping reviews (PRISMA-ScR): checklist and explanation. Ann Intern Med. 2018;169: 467-473. https://doi.org/10.7326/M18-0850

21. Arksey, H. & O'Malley, L. Scoping studies: towards a methodological framework. Int J Soc Res Method. 2005;8(1): 19-32. https://doi.org/10.1080/1364557032000119616

22. Page, M.J, McKenzie, J.E., Bossuyt, P.M., Boutron, I., Hoffmann, T.C., Mulrow, C.D. et al. The PRISMA 2020 statement: an updated guideline for reporting systematic reviews. BMJ. 2021;372(71). https://doi.org/10.1136/bmj.n71

23. Hong, Q.N., Pluye, P., Fábregues, S. et al. Mixed methods appraisal tool (MMAT)

For peer review only

version 2018. User guide. Registration of copyright (#1148552), Canadian intellectual property office, Industry Canada; 2018.

For peer review only

For peer review only - http://bmjopen.bmj.com/site/about/guidelines.xhtml

For peer review only

For peer review only - http://bmjopen.bmj.com/site/about/guidelines.xhtml

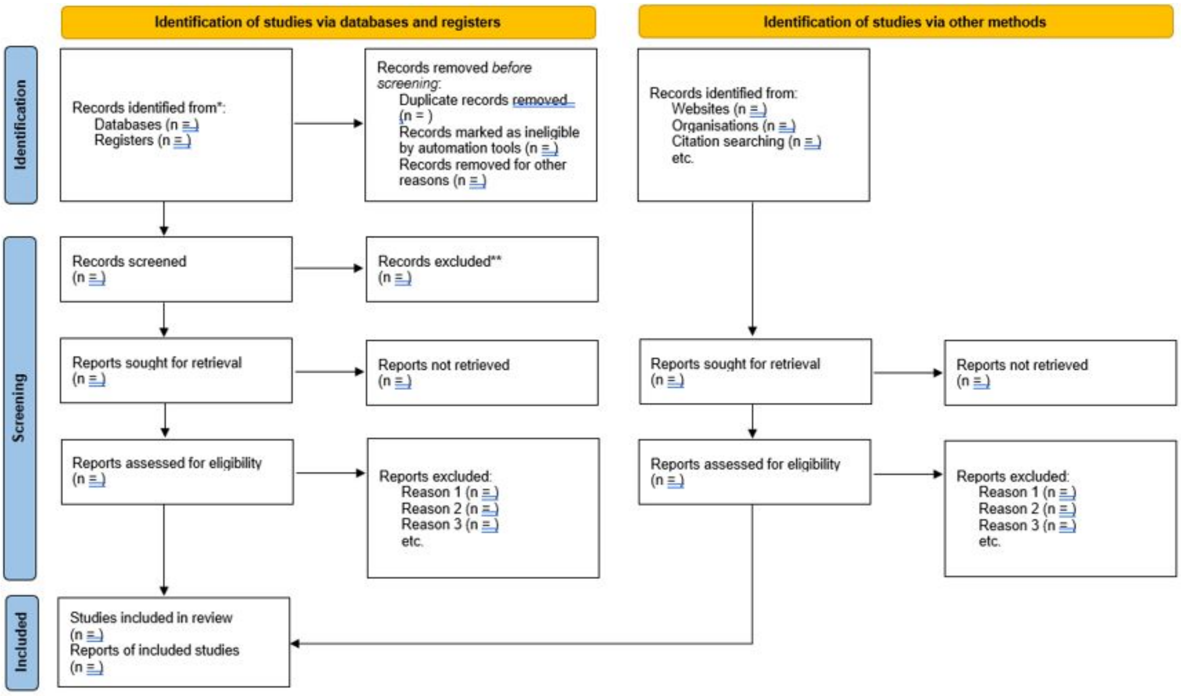
Identification of studies via databases and registers
Identification of studies via other methods
Identification
Records identified from*:
Databases (n =)
Registers (n =)
Records removed before screening:
Duplicate records removed (n =)
Records marked as ineligible by automation tools (n =)
Records removed for other reasons (n =)
Records identified from:
Websites (n =)
Organisations (n =)
Citation searching (n =)
etc.
Screening
Records screened
(n =)
Records excluded**
(n =)
Reports sought for retrieval
(n =)
Reports not retrieved
(n =)
Reports sought for retrieval
(n =)
Reports not retrieved
(n =)
Reports assessed for eligibility
(n =)
Reports excluded:
Reason 1 (n =)
Reason 2 (n =)
Reason 3 (n =)
etc.
Reports assessed for eligibility
(n =)
Reports excluded:
Reason 1 (n =)
Reason 2 (n =)
Reason 3 (n =)
etc.
Included
Studies included in review
(n =)
Reports of included studies
(n =)

Supplemental table 1. Data charting table

Authors	Year of publication	Country	Population	Setting	Aim	Design	Discipline	Quality	RQ1	RQ2	RQ3	RQ4	RQ5

For peer review only

PRISMA-P (Preferred Reporting Items for Systematic review and Meta-Analysis Protocols) 2015 checklist: recommended items to address in a systematic review protocol*

Section and topic	Item No	Checklist item
**ADMINISTRATIVE INFORMATION**		
Title:		
Identification	1a	Identify the report as a protocol of a systematic review **YES page 1**
Update	1b	If the protocol is for an update of a previous systematic review, identify as such **NO**
Registration	2	If registered, provide the name of the registry (such as PROSPERO) and registration number **NO**
Authors:		
Contact	3a	Provide name, institutional affiliation, e-mail address of all protocol authors; provide physical mailing address of corresponding author **YES page 1**
Contributions	3b	Describe contributions of protocol authors and identify the guarantor of the review **YES page 15**
Amendments	4	If the protocol represents an amendment of a previously completed or published protocol, identify as such and list changes; otherwise, state plan for documenting important protocol amendments **NO**
Support:		
Sources	5a	Indicate sources of financial or other support for the review **YES page 15**
Sponsor	5b	Provide name for the review funder and/or sponsor **YES page 15**
Role of sponsor or funder	5c	Describe roles of funder(s), sponsor(s), and/or institution(s), if any, in developing the protocol **NO**
**INTRODUCTION**		
Rationale	6	Describe the rationale for the review in the context of what is already known **YES page 4-5**
Objectives	7	Provide an explicit statement of the question(s) the review will address with reference to participants, interventions, comparators, and outcomes (PICO) **YES page 5, 7**
**METHODS**		
Eligibility criteria	8	Specify the study characteristics (such as PICO, study design, setting, time frame) and report characteristics (such as years considered, language, publication status) to be used as criteria for eligibility for the review **YES page 7, 9**
Information sources	9	Describe all intended information sources (such as electronic databases, contact with study authors, trial registers or other grey literature sources) with planned dates of coverage **YES page 6,7,8**
Search strategy	10	Present draft of search strategy to be used for at least one electronic database, including planned limits, such that it could be repeated **YES page 7**
Study records:		
Data management	11a	Describe the mechanism(s) that will be used to manage records and data throughout the review **YES page 11, 12**

For peer review only

Selection process	11b	State the process that will be used for selecting studies (such as two independent reviewers) through each phase of the review (that is, screening, eligibility and inclusion in meta-analysis) **YES page 11,12**
Data collection process	11c	Describe planned method of extracting data from reports (such as piloting forms, done independently, in duplicate), any processes for obtaining and confirming data from investigators **YES page 11,12,13**
Data items	12	List and define all variables for which data will be sought (such as PICO items, funding sources), any pre-planned data assumptions and simplifications **YES page 11,12**
Outcomes and prioritization	13	List and define all outcomes for which data will be sought, including prioritization of main and additional outcomes, with rationale **YES page 11,12**
Risk of bias in individual studies	14	Describe anticipated methods for assessing risk of bias of individual studies, including whether this will be done at the outcome or study level, or both; state how this information will be used in data synthesis **NO**
Data synthesis	15a	Describe criteria under which study data will be quantitatively synthesised **NO**
	15b	If data are appropriate for quantitative synthesis, describe planned summary measures, methods of handling data and methods of combining data from studies, including any planned exploration of consistency (such as I^2, Kendall's τ) **NO**
	15c	Describe any proposed additional analyses (such as sensitivity or subgroup analyses, meta-regression) **NO**
	15d	If quantitative synthesis is not appropriate, describe the type of summary planned **YES page 13,14**
Meta-bias(es)	16	Specify any planned assessment of meta-bias(es) (such as publication bias across studies, selective reporting within studies) **NO**
Confidence in cumulative evidence	17	Describe how the strength of the body of evidence will be assessed (such as GRADE) **NO**

*** It is strongly recommended that this checklist be read in conjunction with the PRISMA-P Explanation and Elaboration (cite when available) for important clarification on the items. Amendments to a review protocol should be tracked and dated.** **The copyright for PRISMA-P (including checklist) is held by the PRISMA-P Group and is distributed under a Creative Commons Attribution Licence 4.0.**

From: Shamseer L, Moher D, Clarke M, Ghersi D, Liberati A, Petticrew M, Shekelle P, Stewart L, PRISMA-P Group. Preferred reporting items for systematic review and meta-analysis protocols (PRISMA-P) 2015: elaboration and explanation. BMJ. 2015 Jan 2;349(jan02 1):g7647.

For peer review only

BMJ Open

Psychiatric inpatient care for persons with dissociative identity disorder: A scoping review protocol

Journal:	*BMJ Open*
Manuscript ID	bmjopen-2023-079207.R1
Article Type:	Protocol
Date Submitted by the Author:	31-Jan-2024
Complete List of Authors:	Söderberg, Anja; Lulea University of Technology Department of Health Education and Technology,<br>Lindgren, Britt-Marie; Umea Universitet, Nursing<br>Ejneborn-Looi, Git-Marie; Luleå University of Technology, Department of Health Sciences<br>Bäckström, Josefin; Lulea University of Technology Department of Health Education and Technology<br>Gabrielsson, Sebastian; Luleå University of Technology, Department of Health Sciences
**Primary Subject Heading**:	Nursing
Secondary Subject Heading:	Mental health
Keywords:	MENTAL HEALTH, PSYCHIATRY, Hospitals, Hospitalization

SCHOLARONE™
Manuscripts

Psychiatric inpatient care for persons with dissociative identity disorder: A scoping review protocol

Anja Söderberg[1] MSc, PhD Student, RN anja.soderberg@ltu.se

Britt-Marie Lindgren[2] PhD, Professor, RN britt-marie.lindgren@umu.se

Git-Marie Ejneborn Looi[1] PhD, Senior Lecturer, RN git-marie.ejneborn-looi@ltu.se

Josefin Bäckström[1,3] PhD, Senior Lecturer, RN josefin.backstrom@ltu.se

Sebastian Gabrielsson1 PhD, Associate Professor, RN sebastian.gabrielsson@ltu.se

[1]Department of Health, Education and Technology, Luleå University of Technology

[2]Department of Nursing, Umeå University

[3] Department of Medical Sciences, Psychiatry, Uppsala University

Corresponding author:

Anja Söderberg

Department of Health, Education and Technology

Luleå University of Technology

971 87 Luleå

Sweden

anja.soderberg@ltu.se

For peer review only

Abstract

Introduction

Psychiatric inpatient care is often characterized by high pressure and thresholds for admission, brief periods of care and limited time for caring activities. Dissociative identity disorder is a contested diagnosis and persons with dissociative identity disorder are at risk of not receiving adequate support when cared for in psychiatric inpatient care. Because the limited literature addressing the topic includes no overview on how persons with dissociative identity disorder are cared for in psychiatric inpatient settings, the aim of this scoping review is to map the area of knowledge on psychiatric inpatient care for persons experiencing dissociative identity disorder. This scoping review will provide an overview with the possibility to elucidate gaps in the evidence base and needs for future research on psychiatric inpatient care for persons experiencing dissociative identity disorder.

Methods and analysis

This scoping review will follow Preferred Reporting Items for Systematic Review and Meta-Analysis for Scoping Reviews and steps 1-5 described in established method for scoping reviews: identifying research question, identifying relevant studies, study selection, charting the data and collating, summarizing and reporting results.

Ethics approval

Not applicable

Dissemination

This scoping review will be submitted for publication in an international, peer-reviewed journal.

For peer review only

For peer review only - http://bmjopen.bmj.com/site/about/guidelines.xhtml

Keywords

Psychiatric inpatient care, dissociative identity disorder, DID, scoping review

Strengths and limitations

- This proposed scoping review aims to map the area of knowledge broadly, encompassing various types of publications.
- Quality assessment will be integral to the data charting process, contributing to its strength.
- A limitation of this review is that it will not synthesize the findings.

Introduction

Persons with experience of dissociative identity disorder (DID) and other severe dissociative states following trauma experience represent a vulnerable group in psychiatric inpatient care (PIC). This calls for contemporary, personcentered approaches to care that prevent retraumatization and further harm [1]. In those efforts, awareness of the importance of trauma-informed care (TIC) in psychiatric inpatient settings has increasingly been raised [2, 3, 4, 5, 6]. The focus for this proposed scoping review is to provide an overview of what is known about PIC for persons experiencing DID.

Background

PIC is a form of care in which the person is cared for 24 hours a day and the daily activities are regulated by staff [7]. Following the widespread deinstitutionalization in the second half

For peer review only - http://bmjopen.bmj.com/site/about/guidelines.xhtml

of the 20th century, major changes have been made in the organization of psychiatric care around the world [8], such that PIC is no longer the dominant form of care [7]. Access to PIC varies worldwide, from less than two to over 25 hospital beds per 100 000 population [9]. McCrae [10] argues that along with the organizational changes, the view on PIC has also shifted to the idea that PIC in itself is something bad that should be avoided at all costs. Beyond that, PIC settings are often characterized by high pressure and thresholds for admission, brief periods of care and limited time for caring activities [10]. Even so, PIC is considered to be necessary for persons with acute psychiatric problems [11] and PIC of good quality is described to be based on relationships and moreover to be caring, person centered and recovery oriented [12]. Despite recommended models, including TIC, developed to improve the quality of care in PIC [2, 3, 4, 5, 6], PIC facilities struggle to embrace a coherent vision and goal for the delivery of care [13].

Dissociative states, among which DID is considered to be the most severe type [14], include symptoms of depersonalization, derealization [15], flashbacks, nightmares and switching between separate parts of the identity through compartmentalization [16]. DID is defined as a complex yet valid diagnosis that can be distinguished from other disorders by assessing identity alteration and amnesia, and is typically associated with severe childhood trauma [17]. Stigma is recognized to affect persons with dissociative states, who may be undiagnosed for several years and feel shame about their experiences of dissociation [14]. DID is also known to be a controversial and questioned diagnosis [14, 16, 18, 19] with symptoms that can be misinterpreted as better known diagnosis such as schizophrenia or personality syndrome [14, 16, 19] and the person with DID is at risk of not getting adequate treatment and being met with disbelief [1, 19] and a lack of knowledge [20].

Persons experiencing DID are at great risk of self injury and suicide attempts [1, 21, 22, 23, 24, 25] and may require PIC in order to ensure their personal safety and a stabilized wellbeing

For peer review only - http://bmjopen.bmj.com/site/about/guidelines.xhtml

[14]. There is reason to believe that people with DID would benefit from contemporary developments of PIC, including personcentered, recovery-oriented and traumainformed approaches to care [cf. 1]. To date, research on PIC for persons with DID is limited and without any coherent description on the experiences and impact of PIC for persons with DID cared for in PIC. In response, this proposed scoping review will provide an overview with the possibility to elucidate gaps in the evidence base and needs for future research. Due to the relatively unexplored area, a descriptive design is motivated and this proposed scoping review is aiming to map the area of knowledge on psychiatric inpatient care for persons with DID, striving to be as comprehensive as possible in order to identify all relevant literature regardless of study design. The method of scoping review is suitable due to its ability to address broad research questions, map relevant literature and to elucidate the field of interest [cf 26].

Methods and analysis

This scoping review protocol follows Preferred Reporting Items for Systematic Review and Meta-Analysis Protocols checklist (PRISMA-P) [cf 27] and the proposed scoping review will follow Preferred Reporting Items for Systematic Review and Meta-Analysis for Scoping Reviews (PRISMA-ScR) [cf 28].

Step 1-5 in the established method for scoping reviews described by Arksey and O'Malley [26] will be performed as described in what follows:

Identifying research questions

This proposed scoping review aims to map the area of knowledge on PIC for persons with DID. In specific, the review seeks to explore the following research questions (RQ):

- RQ1: How is DID conceptualized in the context of PIC?

For peer review only - http://bmjopen.bmj.com/site/about/guidelines.xhtml

- RQ2: Why are persons with DID subject to PIC?
- RQ3: What mechanisms of PIC for persons with DID are described?
- RQ4: What experiences of PIC for persons with DID are described?
- RQ5: What outcomes of PIC for persons with DID are described?

  Mechanisms refers to key components of care, including interventions, therapies and treatments expected to influence the outcomes of PIC.

Identifying relevant studies

To be as comprehensive as possible, the search strategy will include both peer reviewed papers published in international scholarly journals indexed in curated databases and grey literature [cf 26]. As some information related to health research questions may only be found in grey literature it is suggested that a broad definition of grey literature should be adopted [29]. For the purpose of this proposed review, grey literature refers to research not intended for publication, or not yet published, in international scholarly journals, i.e. dissertations, thesis, conference publications, unpublished manuscripts, and reports from governmental or non-governmental organizations reporting empirical studies.

<u>Search strategy</u>

The first author will perform all of the described steps of the search strategy.

Curated databases

Consultations with a librarian have been undertaken and pilot searches of the literature have been performed in order to refine the search strategy. The databases PubMed, Cinahl and PsycINFO have been identified as being suitable for this proposed scoping review, related to the research area. Key concepts related to the aim and the research questions have also been identified and will form the search blocks. Key concepts are organized in accordance with the Population-Concept-Context-framework (PCC), a framework for

For peer review only - http://bmjopen.bmj.com/site/about/guidelines.xhtml

scoping reviews recommended by the Joanna Briggs Institute [30]. In the search blocks, subject headings will be used together with related terms in free text, and the Boolean operators ("AND" and "OR") will be used to narrow and expand the search. Each search in the databases will be documented with date, search terms, number of search matches, selected studies and limitations. The search strategy for each chosen database is described in Table 1, followed by an example of a pilot search (Table 2):

Table 1: Search strategy

PCC	PubMed	Cinahl	PsycINFO
**Population:** DID	(dissociative identity disorder [MeSH] OR "multiple personality disorder")	("dissociative identity disorder" OR multiple personality disorder [MH] OR did OR mpd)	(dissociative identity disorder [DE] OR "multiple personality disorder")
**Concept:** Psychiatry	(psychiatry [MeSH] OR psychiatric OR mental health [MeSH] OR "mental illness" OR mental disorders [MeSH])	(psychiatry [MH] OR psychiatric OR mental health OR "mental illness" OR mental disorders [MH])	(psychiatry [DE] OR psychiatric OR mental health [DE] or "mental illness" [DE] OR mental disorders [DE])
**Context:** Inpatient care	(inpatients [MeSH] OR ward OR hospital* OR "acute setting")	(inpatients [MH] OR ward OR hospital* OR "acute setting")	(inpatients OR ward OR hospital* OR "acute setting")

For peer review only

**Table 2: Pilot search**		
PubMed 230104		
*Limitations: Published between 2000-2022		
Search	*Search terms*	*Matches*
S1	exp dissociative identity disorder/	1 186
S2	“multiple personality disorder”.mp	356
S3	S1 or S2	1 218
S4	exp psychiatry/	760 502
S5	psychiatric.mp	937 395
S6	exp mental health/	487 022
S7	“mental illness”.mp	40 185
S8	exp mental disorders/	1 459 534
S9	S4 or S5 or S6 or S7 or S8	2 079 336
S10	exp inpatients/	158 439
S11	ward.mp	101 353
S12	hospital*.mp	6 577 438
S13	“acute setting”.mp	2 637
S14	S10 or S11 or S12 or S13	6 669 493
S15	S3 and S9 and S14	260
S16	S3 and S9 and S14*	120

Additional sources

Additional searches will be performed manually in Google Search, Google Scholar and relevant databases that collect theses. Overlapping search strategies are necessary when aiming for breadth, and utilizing these search engines increases the likelihood of finding up-to-date grey literature. However, this approach may generate a large number of search hits and the searches may not be easily replicable [cf 29]. Therefore, a systematic approach with defined search terms and a strategy for managing a large volume of search hits will be implemented. To further identify relevant studies that may not have been found by performing the search strategy in the electronic databases and the additional searches, the reference lists of selected studies will also be screened. Searches for studies that have cited the selected studies will additionally be performed. Key journals, identified through the selected studies, will be searched manually to identify studies that may have been missed in the searches in the electronic databases, in the screening of reference lists and in the citation searches. Last, searches through existing knowledge and networks

For peer review only - http://bmjopen.bmj.com/site/about/guidelines.xhtml

related to the research area will be performed to find information about, for example, studies that have not yet been published [cf 26].

Study selection

To determine the relevance of the studies found by performing the search strategy the inclusion and exclusion criteria will be applied to all the studies found by performing the search strategy. The aim of the inclusion and exclusion criteria is to keep eligible studies and exclude irrelevant studies [cf 26]. In this proposed scoping review the general inclusion and exclusion criteria will consist of year of publication, language and type of study, whereas other inclusion and exclusion criteria are formulated in accordance with the research questions (Table 3):

For peer review only

Table 3: Inclusion and exclusion criteria		
	**Inclusion**	**Exclusion**
Persons with DID	• Studies focused on persons with DID<br>• Studies with small samples of persons with DID but allow distinguishing persons with DID from the other population studied	• Studies focused on persons with DID and a comorbidity
Psychiatry	• Studies focused on psychiatric care	
Inpatient care	• Studies focused on any context in which the person is cared for 24 hours a day<br>• Studies with small samples of inpatient care setting but allow distinguishing the inpatient care setting from other settings studied	
Year of publication	• 2000 - 2023	
Language	• English<br>• Swedish<br>• Norwegian<br>• Danish	
Type of study	• Any paper reporting an original study<br>• Internationally published studies<br>• Grey literature formed as dissertations, thesis, conference publications, unpublished manuscripts and reports from governmental and non-governmental organizations reporting empirical studies	• Editorials<br>• Discursive papers<br>• Literature reviews

For peer review only - http://bmjopen.bmj.com/site/about/guidelines.xhtml

For peer review only

Regarding year of publication, a longer period of time is chosen in order to gather enough data and has been considered in relation to the relatively unexplored area. The limit of the year 2000 has been set due to the organization of PIC after the deinstitutionalization. In addition to English, Swedish, Norwegian, and Danish have been chosen for language considerations, as the authors are Swedish and are proficient in these Scandinavian languages.

To apply the inclusion and exclusion criteria to all of the identified studies, a systematic approach will be undertaken. The screening of all studies will follow a combined template for screening and charting (Table 4).

For peer review only

For peer review only - http://bmjopen.bmj.com/site/about/guidelines.xhtml

Table 4: Screening and charting template	
**Step 1: Title and abstract screening**	
1. Is this title and abstract written in English/Swedish/Norwegian/Danish?	Yes/No
2. Does it seemingly address DID in PIC?	Yes/No
**Step 2: Full-text screening**	
1. Is there any reason this article should be excluded?	Yes/No
If yes:<br>What is the reason for excluding?	1. Not in English/Swedish/Norwegian/Danish<br>2. Not published between 2000 – 2023<br>3. Not focus on DID<br>4. Not focus on PIC<br>5. No full text available despite efforts to retrieve
If no:<br>What is the data charting information?	Use the data charting table
2. Are there descriptions on how DID is conceptualized in the context of PIC (RQ1)?	Yes/No
If yes, describe how:	
3. Are there descriptions of why persons with DID are subject to PIC (RQ2)?	Yes/No
If yes, describe how:	
4. Are there descriptions of mechanisms of PIC for persons with DID (RQ3)?	Yes/No
If yes, describe how:	
5. Are there descriptions of experiences of PIC for persons with DID (RQ4)?	Yes/No
If yes, describe how:	
6. Are there descriptions of outcomes of PIC for persons with DID (RQ5)?	Yes/No
If yes, describe how:	

The results of the searches from each database will be downloaded in the screening tool Rayyan to support a systematic screening. A first selection of studies will be made through applying the inclusion criteria on the title and abstract of all the studies. The next step will include reading the studies in full in order to make a final decision whether to include or

For peer review only - http://bmjopen.bmj.com/site/about/guidelines.xhtml

exclude, which is required since abstracts can't be expected to represent the full study or to grasp the full scope of the study [cf 26]. Both steps of the study selection will include two authors independently screening the studies. If disagreements whether to include or exclude the two authors will discuss and try to reach consensus, and if needed a third author will be involved to resolve discrepancies. The process of selecting eligible studies will follow the Preferred Reporting Items for Systematic Review and Meta-Analysis (PRISMA) flow diagram (Figure 1) [31]:

Figure 1. PRISMA flow diagram

Charting the data

The combined template for screening and charting (Table 4) will also include steps for charting the data and will be used to support a systematic approach. Data from the selected studies will then be charted according to the research questions. The charted data will be transferred to a data charting table (Supplemental table 1) that includes the following information:

- Authors
- Year of publication
- Country
- Population
- Setting
- Aim
- Design
- Discipline
- Quality
- RQ1

For peer review only

For peer review only - http://bmjopen.bmj.com/site/about/guidelines.xhtml

- RQ2
- RQ3
- RQ4
- RQ5

The charted data will then be used as the foundation for analysis [cf 26]. Two authors will chart the data independently and disagreements will be resolved through discussion until consensus is reached.

Quality assessment

Because scoping reviews generally seek comprehensiveness, breadth and inclusion, a quality assessment is not included in the description of a scoping review which is acknowledged as a limitation of the method [26]. However, in this proposed scoping review a quality assessment will be performed as a part of the data charting in order to also provide an overview on the quality of the existing research. The Mixed Method Appraisal Tool [32] will be used for the quality appraisal of the found studies. This tool is developed to be used in reviews containing qualitative, quantitative and mixed method studies [32], why it's chosen for the quality appraisal of this review. Two authors will independently perform the quality appraisal and disagreements will be discussed until consensus is reached. A third author will be involved in the process to resolve discrepancies if needed.

Collating, summarizing and reporting the results

The final part of this proposed scoping review will provide an overview of the research by collating, summarizing and reporting results. One part of this analysis will be a basic numerical analysis of the included studies, meaning the data presented in the data charting table (Supplemental table 1), in order to bring light to dominant areas of research by to country, study population, setting, aims, designs and disciplines. The second part of the

For peer review only - http://bmjopen.bmj.com/site/about/guidelines.xhtml

analysis will be to organize the data thematically according to the research questions. A clear reporting strategy will be undertaken in order to determine potential bias, which is why a consistent approach based on the combined template for screening and charting (Table 4), also covering the research questions, will be used. The next step will be to make comparisons, identify contradictions, identify gaps in the evidence base and suggest topics for future research [cf 26]. All authors will be involved in the final discussion about the collated, summarized and reported results.

Patient and public involvement

A non-governmental organization advocating the interests of persons experiencing DID, including people with personal experience of DID, have been engaged in the development of the research questions and the design of the review. Members of the board were invited to take part of the research plan and participated in several discussions on the research plan together with the research team. No major changes were made after discussions with the non-governmental organization. The findings from the review will be communicated with representatives of the organization.

List of abbreviations

**DID**	Dissociative identity disorder
**PIC**	Psychiatric inpatient care
**PRISMA**	Preferred reporting items for systematic review and meta-analysis
**PRISMA-P**	Preferred reporting items for systematic review and meta-analysis protocols

For peer review only - http://bmjopen.bmj.com/site/about/guidelines.xhtml

**PRISMA-ScR**	Preferred reporting items for systematic review and meta-analysis for scoping reviews
**TIC**	Traumainformed care

Ethics approval

Not applicable

Dissemination

This proposed scoping review will be submitted for publication in an international, peer-reviewed journal

Authors' contributions

AS, SG, BL and GEL have made substantial contributions to the conception and the design. AS and SG have drafted the work and AS, SG, BL, GEL and JB have substantively revised it. Each author has approved to the submitted version and have agreed to be personally accountable and to ensure that questions related to the accuracy or integrity of any part of the work, even ones in which the author was not personally involved, are appropriately investigated, resolved, and the resolution documented in the literature.

Funding

This research is funded by Luleå university of technology

For peer review only - http://bmjopen.bmj.com/site/about/guidelines.xhtml

Competing interests statement

The authors declare no conflict of interest

Data availability statement

The authors confirm that the data supporting the findings of this study are available within the article and/or its supplementary materials.

For peer review only

For peer review only - http://bmjopen.bmj.com/site/about/guidelines.xhtml

References

1. Parry, S., Lloyd, M. & Simpson, J. Experiences of therapeutic relationships on hospital wards, dissociation, and making connections. J Trauma Dissociatio. 2017;18(4): 544-558. https://doi.org/10.1080/15299732. 2016.1241852

2. Isobel, S. 'Because that's the way it's always been done': Reviewing the nurse-initiated rules in a mental health unit as a step toward trauma-informed care. Issues in Ment Health N. 2015;36: 272-278. https://doi.org/10.3109/01612840.2014.982842

3. Isobel, S. & Edwards, C. Using trauma informed care as a nursing model of care in an acute inpatient mental health unit: A practice development process. Int J Ment Health Nu. 2016. https://doi.org/10.1111/inm.12236

4. O'Dwyer, C., Tarzia, L., Fernbacher, S. & Hegarty, K. Health professionals' experiences of providing trauma-informed care in acute psychiatric inpatient settings: A scoping review. Trauma Violence Abus. 2021;22(5): 1057-1067. https://doi.org/10.1177/1524838020903064

5. Muskett, C. Trauma-informed care in inpatient mental health settings: A review of the literature. Int J Ment Hlt Nu. 2014;23: 51-59. https://doi.org/10.1111/inm.12012

For peer review only - http://bmjopen.bmj.com/site/about/guidelines.xhtml

6. Wilson, A., Hutchinson, M. & Hurley, J. Literature review of trauma-informed care: Implications for mental health nurses working in acute inpatient settings in Australia. Int J Ment Hlt Nu. 2017;26: 326-343. https://doi.org/10.1111/inm.12344

7. Topor, A., Andersson, G., Bülow, P., Stefansson, C. G., & Denhov, A. After the asylum? The new institutional landscape. Community Ment Hlt J. 2016;52(6): 731-737

8. Bergmark, M., Bejerholm, U. & Markström, U. (2017). Policy changes in community mental health: Interventions and strategies used in Sweden over 20 years. Soc Policy Admin. 2017;51: 95–113. https://doi.org/10.1111/spol.1275

9. World Health Organization. Mental Health Atlas 2020. World Health Organization; 2021.

10. McCrae, N. Lessons from a lost world: Messages from the mental hospital era. Editorial. J Adv Nurs. 2017;74(6): 1226-1228. https://doi.org/10.1111/jan.13467

11. Jacob, K. S., Sharan, P., Mirza, I., Garrido-Cumbrera, M., Seedat, S., Mari, J. J., Sreenivas, V. & Saxena, S. (2007). Mental health systems in countries: where are we now? Lancet. 2007; 370(9592), 1061-1077

For peer review only

For peer review only - http://bmjopen.bmj.com/site/about/guidelines.xhtml

12. Gabrielsson, S., Tuvesson, H., Wiklund Gustin, L. & Jormfeldt, J. Positioning psychiatric and mental health nursing as a transformative force in health care. Issues Ment Health N. 2020;41(11): 976-984. https://doi.org/10.1080/01612840.2020.1756009

13. Salberg, J., Bäckström, J., Röing, M., & Öster, C. Ways of understanding nursing in psychiatric inpatient care–A phenomenographic study. J Nurs Manage. 2019; 27(8): 1826-1834.

14. Snyder, B.L. Recognizing & treating dissociative disorders: the nurse's role in supporting patients and their families. American Nurse Journal. 2021;16(9): 2-5.

15. Hill, S.B., Wolff, J.D., Bigony, C.E., Winternitz, S.R., Ressler, K.J., Kaufman, M.L. & Lebois, L.A.M. Dissociative subtype of posttraumatic stress disorder in women in partial and residential levels of psychiatric care. J Trauma Dissociation. 2020; 21(3): 305-318. https://doi.org/10.1080/15299732.2019.1678214

16. McAllister, M. M. Dissociative identity disorder: A literature review. J Psychiatr Ment Hlt. 2000;7(1): 25-33. https://doi.org/10.1046/j.1365-2850.2000.00259

17. Dorahy, M., Brand, B., Sar, V., Krüger, C., Stavropoulos, P., Martinez-Taboas, A., Lewis-Fernández, R. & Middleton, W. Dissociative identity disorder: an empirical

For peer review only - http://bmjopen.bmj.com/site/about/guidelines.xhtml

overview. Aust N Z J Psychiatry. 2014;48(5): 402-417. https://doi.org/10.1177/0004867414527523

18. Stickley, T., & Nickeas, R. Becoming one person: Living with dissociative identity disorder. J Psychiatr Ment Hlt. 2006;13(2): 180-187.

19. Tomlinson, K. & Baker, C. Women's auto/biography and dissociative identity disorder: implications for mental health practice. Journal of Medical Humanit. 2019;40: 365-387. https://doi.org/10.1007/s10912-017-9471-3

20. Kumar, S., Brand, B. & Courtois, C. The need for trauma training: clinicians' reactions to training on complex trauma. Psychol Trauma. 2022;14(8): 1387-1394. https://doi.org/10.1037/tra0000515

21. Brand, B., Classen, C., Lanins, R., Loewenstein, R., McNary, R., Pain, C. & Putnam, F. A naturalistic study of dissociative identity disorder and dissociative disorder not otherwise specified patients treated by community clinicians. Psychol Trauma. 2009;1(2): 153-171. https://doi.org/10.1037/a0016210

22. Brand, B., McNary, S., Myrick, A., Classen, C., Lanius, R., Loewenstein, R., Pain, C. & Putnamn, F. A longitudinal naturalistic study of patients with dissociative disorders

For peer review only - http://bmjopen.bmj.com/site/about/guidelines.xhtml

treated by community clinicians. Psychol Trauma. 2013;5(4): 301-308. https://doi.org/10.1037/a0027654

23. Brand, B., Schielke, H., Putnam, K., Putnam, F., Loewenstein, R., Myrick, A., Jepsen, E., Langeland, W., Steele, K., Classen, C. & Lanius, R. An online educational program for individuals with dissociative disorders and their clinicians: 1-Year and 2-year follow-up. J Trauma Stress. 2019; 32: 156-166. https://doi.org/10.1002/jts.22370

24. Foote, B., Smolin, Y., Neft, D.I. & Lipshitz, D. Dissociative disorders and suicidality in psychiatric outpatients. J Nerv Ment Dis. 2008;196(1): 29-36. https://doi.org/10.1097/NMD.0b013e31815fa4e7

25. Nester, M., Brand, B., Schielke, H. & Kumar, S. An examination of the relations between emotion dysregulation, dissociation, and self-injury among dissociative disorder patients. Eur J Psychotraumatol. 2022;13(1). https://doi.org/10.1080/20008198.2022.2031592

26. Arksey, H. & O'Malley, L. Scoping studies: towards a methodological framework. Int J Soc Res Method. 2005;8(1): 19-32. https://doi.org/10.1080/1364557032000119616

For peer review only - http://bmjopen.bmj.com/site/about/guidelines.xhtml

27. Moher, D., Stewart, L. & Shekelle, P. Implementing PRISMA-P: recommendations for prospective authors. Syst Rev. 2016;5(15). https://doi.org/10.1186/s13643-016-0191-y

28. Tricco, A.C., Lillie, E., Zarin, W. et al. PRISMA extension for scoping reviews (PRISMA-ScR): checklist and explanation. Ann Intern Med. 2018;169: 467-473. https://doi.org/10.7326/M18-0850

29. Adams, J., Hillier-Brown, F.C., Moore, H.J., Lake, A.A., Araujo-Soares, V., White, M. & Summerbell, C. Searching and synthesising 'grey literature' and 'grey information' in public health: critical reflections on three case studies. Syst Rev. 2016;5(164). https://doi.org/10.1186/s13643-016-0337-y

30. Aromataris E. & Munn, Z. (Ed.). JBI Manual for Evidence Synthesis. JBI, 2020.

31. Page, M.J, McKenzie, J.E., Bossuyt, P.M., Boutron, I., Hoffmann, T.C., Mulrow, C.D. et al. The PRISMA 2020 statement: an updated guideline for reporting systematic reviews. BMJ. 2021;372(71). https://doi.org/10.1136/bmj.n71

32. Hong, Q.N., Pluye, P., Fábregues, S. et al. Mixed methods appraisal tool (MMAT) version 2018. User guide. Registration of copyright (#1148552), Canadian intellectual property office, Industry Canada; 2018.

For peer review only - http://bmjopen.bmj.com/site/about/guidelines.xhtml

For peer review only

For peer review only - http://bmjopen.bmj.com/site/about/guidelines.xhtml

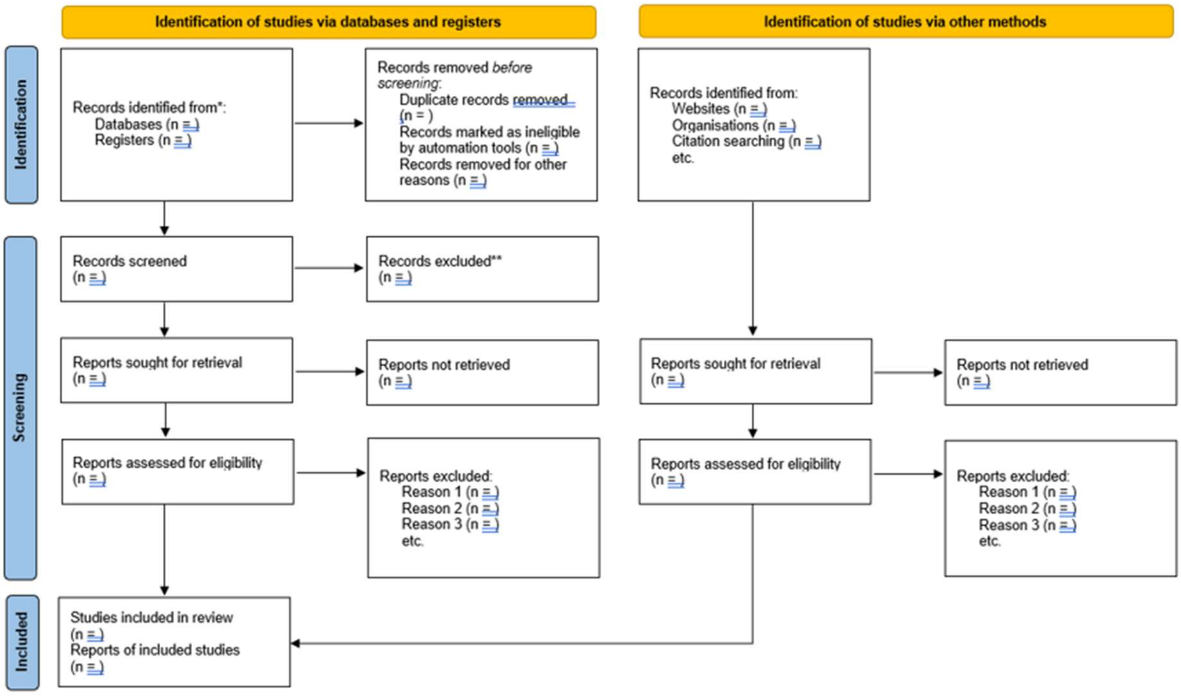

For peer review only

Supplemental table 1. Data charting table

Authors	Year of publication	Country	Population	Setting	Aim	Design	Discipline	Quality	RQ1	RQ2	RQ3	RQ4	RQ5

For peer review only

PRISMA-P (Preferred Reporting Items for Systematic review and Meta-Analysis Protocols) 2015 checklist: recommended items to address in a systematic review protocol*

Section and topic	Item No	Checklist item
**ADMINISTRATIVE INFORMATION**		
Title:		
Identification	1a	Identify the report as a protocol of a systematic review **YES page 1**
Update	1b	If the protocol is for an update of a previous systematic review, identify as such **NO**
Registration	2	If registered, provide the name of the registry (such as PROSPERO) and registration number **NO**
Authors:		
Contact	3a	Provide name, institutional affiliation, e-mail address of all protocol authors; provide physical mailing address of corresponding author **YES page 1**
Contributions	3b	Describe contributions of protocol authors and identify the guarantor of the review **YES page 15**
Amendments	4	If the protocol represents an amendment of a previously completed or published protocol, identify as such and list changes; otherwise, state plan for documenting important protocol amendments **NO**
Support:		
Sources	5a	Indicate sources of financial or other support for the review **YES page 15**
Sponsor	5b	Provide name for the review funder and/or sponsor **YES page 15**
Role of sponsor or funder	5c	Describe roles of funder(s), sponsor(s), and/or institution(s), if any, in developing the protocol **NO**
**INTRODUCTION**		
Rationale	6	Describe the rationale for the review in the context of what is already known **YES page 4-5**
Objectives	7	Provide an explicit statement of the question(s) the review will address with reference to participants, interventions, comparators, and outcomes (PICO) **YES page 5, 7**
**METHODS**		
Eligibility criteria	8	Specify the study characteristics (such as PICO, study design, setting, time frame) and report characteristics (such as years considered, language, publication status) to be used as criteria for eligibility for the review **YES page 7, 9**
Information sources	9	Describe all intended information sources (such as electronic databases, contact with study authors, trial registers or other grey literature sources) with planned dates of coverage **YES page 6,7,8**
Search strategy	10	Present draft of search strategy to be used for at least one electronic database, including planned limits, such that it could be repeated **YES page 7**
Study records:		
Data management	11a	Describe the mechanism(s) that will be used to manage records and data throughout the review **YES page 11, 12**

For peer review only

Selection process	11b	State the process that will be used for selecting studies (such as two independent reviewers) through each phase of the review (that is, screening, eligibility and inclusion in meta-analysis) **YES page 11,12**
Data collection process	11c	Describe planned method of extracting data from reports (such as piloting forms, done independently, in duplicate), any processes for obtaining and confirming data from investigators **YES page 11,12,13**
Data items	12	List and define all variables for which data will be sought (such as PICO items, funding sources), any pre-planned data assumptions and simplifications **YES page 11,12**
Outcomes and prioritization	13	List and define all outcomes for which data will be sought, including prioritization of main and additional outcomes, with rationale **YES page 11,12**
Risk of bias in individual studies	14	Describe anticipated methods for assessing risk of bias of individual studies, including whether this will be done at the outcome or study level, or both; state how this information will be used in data synthesis **NO**
Data synthesis	15a	Describe criteria under which study data will be quantitatively synthesised **NO**
	15b	If data are appropriate for quantitative synthesis, describe planned summary measures, methods of handling data and methods of combining data from studies, including any planned exploration of consistency (such as I^2, Kendall's τ) **NO**
	15c	Describe any proposed additional analyses (such as sensitivity or subgroup analyses, meta-regression) **NO**
	15d	If quantitative synthesis is not appropriate, describe the type of summary planned **YES page 13,14**
Meta-bias(es)	16	Specify any planned assessment of meta-bias(es) (such as publication bias across studies, selective reporting within studies) **NO**
Confidence in cumulative evidence	17	Describe how the strength of the body of evidence will be assessed (such as GRADE) **NO**

*** It is strongly recommended that this checklist be read in conjunction with the PRISMA-P Explanation and Elaboration (cite when available) for important clarification on the items. Amendments to a review protocol should be tracked and dated. The copyright for PRISMA-P (including checklist) is held by the PRISMA-P Group and is distributed under a Creative Commons Attribution Licence 4.0.**

From: Shamseer L, Moher D, Clarke M, Ghersi D, Liberati A, Petticrew M, Shekelle P, Stewart L, PRISMA-P Group. Preferred reporting items for systematic review and meta-analysis protocols (PRISMA-P) 2015: elaboration and explanation. BMJ. 2015 Jan 2;349(jan02 1):g7647.

For peer review only
